# *Helicobacter pylori cag* Pathogenicity Island (*cag*PAI) Involved in Bacterial Internalization and IL-8 Induced Responses via NOD1- and MyD88-Dependent Mechanisms in Human Biliary Epithelial Cells

**DOI:** 10.1371/journal.pone.0077358

**Published:** 2013-10-15

**Authors:** Wongwarut Boonyanugomol, Chariya Chomvarin, Chariya Hahnvajanawong, Banchob Sripa, Maria Kaparakis-Liaskos, Richard L. Ferrero

**Affiliations:** 1 Department of Microbiology, Faculty of Medicine, Khon Kaen University, Khon Kaen, Thailand; 2 Department of Pathology, Faculty of Medicine, Khon Kaen University, Khon Kaen, Thailand; 3 Liver Fluke and Cholangiocarcinoma Research Center, Faculty of Medicine, Khon Kaen University, Khon Kaen, Thailand; 4 Centre for Innate Immunity and Infectious Diseases, Monash Institute of Medical Research, Clayton, Victoria, Australia; Veterans Affairs Medical Center (111D), United States of America

## Abstract

*Helicobacter pylori* infection has been proposed to be associated with various diseases of the hepatobiliary tract, including cancer of the bile duct epithelial cells (cholangiocarcinoma, CCA). The ability of *H. pylori* bacteria to cause pathogenic effects in these cells has, however, yet to be investigated. Given that the *cag* pathogenicity island (*cag*PAI) is required for *H. pylori* pathogenesis in gastric epithelial cells, we investigated wild-type and *cag* mutant strains for their ability to adhere, be internalized and induce pro-inflammatory responses in two bile duct epithelial cell lines derived from cases of CCA. The findings from these experiments were compared to results obtained with the well-characterized AGS gastric cancer cell line. We showed that the *cag*PAI encodes factors involved in *H. pylori* internalization in CCA cells, but not for adhesion to these cells. Consistent with previous studies in hepatocytes, actin polymerization and α5β1 integrin may be involved in *H. pylori* internalization in CCA cells. As for AGS cells, we observed significantly reduced levels of NF-κB activation and IL-8 production in CCA cells stimulated with either cagA, *cag*L or *cag*PAI bacteria, when compared with wild-type bacteria. Importantly, these IL-8 responses could be inhibited via either pre-treatment of cells with antibodies to α5β1 integrins, or via siRNA-mediated knockdown of the innate immune signaling molecules, nucleotide oligomerization domain 1 (NOD1) and myeloid differentiation response gene 88 (MyD88). Taken together, the data demonstrate that the *cag*PAI is critical for *H. pylori* pathogenesis in bile duct cells, thus providing a potential causal link for *H. pylori* in biliary tract disease.

## Introduction

The Gram-negative bacterium, *Helicobacter pylori*, is a causative agent of various gastroduodenal diseases, including gastric adenocarcinoma [1]. It has been mooted that *H. pylori* may also play a role in the development of hepatobiliary disease, particularly liver cancer [2–4]. One such disease, cholangiocarcinoma (CCA), is a cancer of bile duct epithelial cells and is highly prevalent in Northeast Thailand [5]. The disease process associated with CCA, however, has yet to be fully elucidated. In a previous report, we showed that *H. pylori* and its pro-oncogenic effector molecule, cagA, were more frequently detected in CCA patients [6,7]. Moreover, the presence of *H. pylori* was associated with biliary inflammation and proliferation when compared with cholelithiasis and in control subjects [6]. We hypothesized that *H. pylori* might be involved in CCA development.

Several virulence factors of *H. pylori* are proposed to play a role in pathogenesis [8]. A major factor is the *cag* pathogenicity island (PAI), which consists of approximately 30 genes, encoding a type 4 secretion system (T4SS), capable of delivering CagA and a bacterial cell wall component, peptidoglycan, into host cells [9]. The T4SS of *H. pylori* forms a pilus-like structure encoded with CagL, which interacts with α5β1 integrin on host cells [10]. CagL interactions with α5β1 integrin are thought to be essential for *H. pylori* pathogenesis [10]. *H. pylori* strains that possess a functional T4SS are more frequently associated with severe inflammation and gastric cancer [9]. 

Host cells possess a variety of putative pathogen recognition molecules (PRMs) capable of modulating both innate and adaptive immune responses through their sensing of conserved microbial components. One of these PRMs, Nucleotide Oligomerization Domain 1 (NOD1), which is known to specifically recognize Gram-negative peptidoglycan, was shown to play a critical role in pro-inflammatory responses to infection by *H. pylori cag*PAI-positive strains [8]. A separate study identified an essential adapter molecule of multiple Toll-like receptor (TLR) pathways, myeloid differentiation response gene 88 (MyD88), as being important for pro-inflammatory host cell signaling to *H. pylori* infection [11]. 

Our previous *in vitro* studies revealed that *H. pylori* induces multiple effects in CCA cell lines, including inflammation (IL-8 production), cell proliferation and apoptosis [12,13]. We also found that at a low multiplicity of infection (MOI=1), *H. pylori* could induce inflammatory and cell proliferative responses in CCA cell lines. This finding suggests that the small numbers of *H. pylori* bacteria that reach the epithelial cells of the hepatobiliary tract may be sufficient to promote inflammation and transformation within this niche; thereby supporting the potential role of the bacterium in the development of hepatobiliary disease [12]. In order to investigate this hypothesis, as well as the possible link between *cag*PAI-positive *H. pylori* strains and CCA, we tested the ability of various *H. pylori* wild-type and isogenic *cag* mutant strains to adhere, invade and induce pro-inflammatory responses in two CCA cell lines. Furthermore, we examined the roles of α5β1 integrin, NOD1 and several TLR family members in these responses and compared the findings with those obtained using a standard gastric epithelial cell line (AGS). We herein elucidate the mechanisms whereby *cag*PAI-positive *H. pylori* induce pro-inflammatory responses in biliary tract epithelial cells, thus providing a potential pathogenic link between the bacterium and hepatobiliary disease.

## Materials and Methods

### Bacterial strains

*H. pylori* wild-type strain 251 [14], cagA (cagA ^-^) [14], *cag*L (this study, generated using the gene deletion strategy described by Gorrell et al. [15]) and *cag*PAI (*cag*PAI^-^) [16] isogenic mutant strains were grown on Oxoid Blood Agar Base No. 2 (Thermo Fisher Scientific, Australia Pty Ltd) supplemented with 5% whole horse blood (Thermo Fisher Scientific, Australia Pty Ltd) overnight at 37°C under microaerobic conditions. *Shigella flexneri* (M90T serotype 5A) was cultured on Trypticase Soy Agar, supplemented with 0.01% Congo red at 37°C.

### Cell Culture

The human cholangiocarcinoma cell lines (KKU-100 and KKU-M156) were obtained from the Liver Fluke and Cholangiocarcinoma Research Center (Khon Kaen University, Thailand) [17–20]. These cells were cultured in Ham F-12 medium supplemented with 10% FBS, streptomycin (100 µg/ml) and penicillin (1 IU/ml) and incubated at 37°C in a 5% CO_2,_ humidified atmosphere. The AGS gastric cancer cell line was cultured as previously described [21]. 

### Adherence and internalization assays

Cells were cultured in 12-well tissue culture plates (1 x 10^5^ cells per well) and allowed to grow overnight. Prior to stimulation, the media was removed and replaced with serum free media, and cells were incubated with *H. pylori* wild type, mutant or *S. flexneri* at an MOI of 1:100 [22], for 6 h. After incubation, the cell culture medium was removed and the treated cells were washed three times with PBS. To determine the number of adherent bacteria, cells were scraped from the tissue culture plates. For the invasion assay, the cell culture medium was removed and extracellular *H. pylori* killed by gentamicin (100 mg/ml) for 6 h. After gentamicin treatment, the treated cells were washed three times with PBS and lysed using 1% saponin for 15 min. Adherent and invasive bacteria, respectively, were estimated by plating of serial dilutions. The number of adherent or invasive bacteria were calculated as percentages of the total number of bacteria added to cells. 

### Inhibition of bacterial internalization by cytochalasin D or α5β1 integrin antibodies

Cells were grown in 12-well tissue culture plates and pre-treated for 30 min with either cytochalasin D (5 μg/ml) (Sigma, St. Louis, MO) or α5β1 integrin antibodies (5 μg/ml) (AIIB2 rat anti-human β_1_integrin, IgG1, BIIG2 rat anti-human α_5_ integrin, IgG2b κ integrin-blocking antibodies, Developmental Studies Hybridoma Bank, University of Iowa, USA) for 1 h at 37°C with 5% CO_2_, as previously described [23]. After treatment, the cells were co-cultured with *H. pylori* wild type, *cag*PAI^-^ or *S. flexneri* at an MOI of 1:100 for 6 h. The numbers of internalized *H. pylori* were determined as described above.

### Detection of NF-κB activation in CCA cells

To measure NF-κB activation, cells were co-cultured with *H. pylori* wild type, cagA ^-^ or *cag*PAI^-^ strains (MOI=1) for 6 h. Phorbol myristate acetate (PMA) was used as the positive control (200 ng/ml). The treated cells were washed with PBS, fixed in 8% (v/v) formaldehyde then permeabilized with absolute methanol for 10 min at -20°C. Cells were washed with PBS and blocked with 5% fetal calf serum (containing 3% Triton X-100) at room temperature for 30 min. The cells were then incubated with rabbit anti-p65 antibody (Santa Cruz, USA) (dilution 1:100) for 1 h at room temperature. After washing three times with PBS, the cells were incubated with goat anti-rabbit conjugated-Alexa 647 (Santa Cruz, USA) for 30 min at room temperature. Cells were again washed three times with PBS and stained with DAPI (Molecular Probes, 1:10,000) for 5 min. Nuclear translocation of p65-containing NF-κB complexes were measured as the intensity of fluorescence within the cell nuclei, using a Cellomic Array Scan^TM^ (Thermo Scientific, USA) machine. Twenty images per well were captured (200X magnification).

### IL-8 Enzyme-linked Immunosorbent Assay (ELISA)

Cells were co-cultured with *H. pylori* wild type, cagA ^-^ or *cag*PAI^-^ strains (MOI=1) for 6 h. To determine the role of α5β1 integrin in IL-8 responses, the cells were pre-treated with combined α5β1 integrin antibodies (5 μg/ml) for 1 h. Cell culture supernatants were collected and IL-8 was quantified by ELISA (BD Bioscience Pharmingen, CA, USA), as per the manufacturer’s instructions. 

### RNA extraction

Cells were co-cultured with *H. pylori* wild type, cagA ^-^ or *cag*PAI^-^ mutant strains (MOI=1) for 6, 12 or 24 h. At each time-point, the cells were washed with PBS and RNA extracted using the PureLink^TM^ RNA purification kit (Life Technologies Corp., USA), according to the manufacturer’s instructions. RNA samples were eluted in 50 μl of elution buffer and stored at -80°C until used.

### qRT-PCR detection of *NOD1*, *TLR2*, *TLR4* and *TLR5* gene expression

RNA (2 μg) was reverse transcribed using SuperScript III^TM^ (Life Technologies Corp., USA), according to the manufacturer’s instructions. Briefly, RNA was added to 20 µl of master mix containing 10 mM dNTP mix, 25 mM MgCl_2_, 0.1 M dithiothreitol (DTT), 40 U RNase inhibitor, 50 μM oligo(dT) and 200 U of Moloney murine leukemia virus reverse transcriptase. cDNA synthesis was performed by incubation at 50°C for 50 min. 

The primers used to amplify *NOD1* [21], β-actin (*ACTB*) [21], *TLR2*, *TLR4* and *TLR5* are listed in Table 1. Each reaction contained 1 μM of forward and reverse primers, 5 μl of SYBR Green PCR master mix (Applied Biosystems, Warrington, UK) and 1 μl of cDNA. Each reaction was made to a final volume of 10 μl with ultrapure distilled water. Polymerase Chain Reactions (PCRs) were performed in an ABI Prism 7700 Sequence Detection System (Applied Biosystems, Victoria, Australia) as follows: 50°C for 2 min, 95°C for 10 min, followed by 40 cycles at 95°C for 15 sec and 60°C for 1 min. The cycle threshold (Ct) values for each gene were normalized to the Ct value for β-actin. The expression levels of each gene were compared to those of control cells.

**Table 1:** Primer sequences used for qRT-PCR.

**genes**	**Primer sequences**		**Refs**
*NOD1*	5'-ACGATGAAGTGGCAGAGAGTT -3		[21]
	5'-GGCAGTCCCCTTAGCTGTGA -3'		
*TLR2*	5'-GCCTCTCCAAGGAAGAATCC -3		This study (unpublished)
	5'-TCCTGTTGTTGGACAGGTCA -3		
*TLR4*	5'-AAGCCGAAAGGTGATTGTTG -3		This study (unpublished)
	5'-CTGAGCAGGGTCTTCTCCAC-3'		
*TLR5*	5'-TGCCTTGAAGCCTTCAGTTATG -3'		This study (unpublished)
	5'-CCAACCACCACCATGATGAG-3'		
*ACTB*	5'-GATGAGATTGGCATGGCTTT -3'		[21]
	5'-CACCTTCACCGTTCCCAGTTT -3'		
*MYD88*	5' -CTCCTCCACATCCCTTCC -3'		[53]
	5' -CCGCACGTTCAAGAACAGAGA -3'		

### siRNA knock-down of *NOD1* and *MYD*88 expression

Pre-designed RNA oligonucleotides for *NOD1* and *MYD*88 were supplied by Ambion (Life Technologies Corp.) with the following siRNA ID numbers: *NOD1* (S20322, 20324) and *MYD*88 (S9138, S9136). siRNA to the human β-defensin 3 (HBD3) gene (*DEFB103*, si04269552, Qiagen) was used as a negative control. Mock transfection control samples comprised Opti-MEM containing lipofectamine 2000 without siRNA. In brief, siRNAs were diluted to a final concentration of 4 μM in Opti-MEM medium containing lipofectamine 2000 (Life Technologies Corp.). These siRNA mixtures were incubated at room temperature for 20 min, then aliquots (100 μl) were added directly into each well of a 24-well plate (in triplicate). Cell suspensions (antibiotic-free) were seeded at a final density of 1 x 10^5^ cells/well into each well containing the appropriate siRNA mixture. After 24 h of incubation, the media was removed and transfected cells were co-cultured with *H. pylori* wild-type or *cag*PAI^-^ strains (MOI=1) for 24 h. Cell culture supernatants were collected to quantify levels of IL-8 by ELISA. Each experiment was performed in triplicate. *NOD1* and *MYD*88 knock-down (KD) was confirmed by qRT-PCR using primers listed in Table 1.

### Statistical Analysis

Data are reported as means ± SEM. Differences between samples were analyzed using the Student’s t test. *p* values < 0.05 were considered significant.

## Results

### *H. pylori* adheres to and is internalized by biliary tract epithelial cells

**Figure 1:**
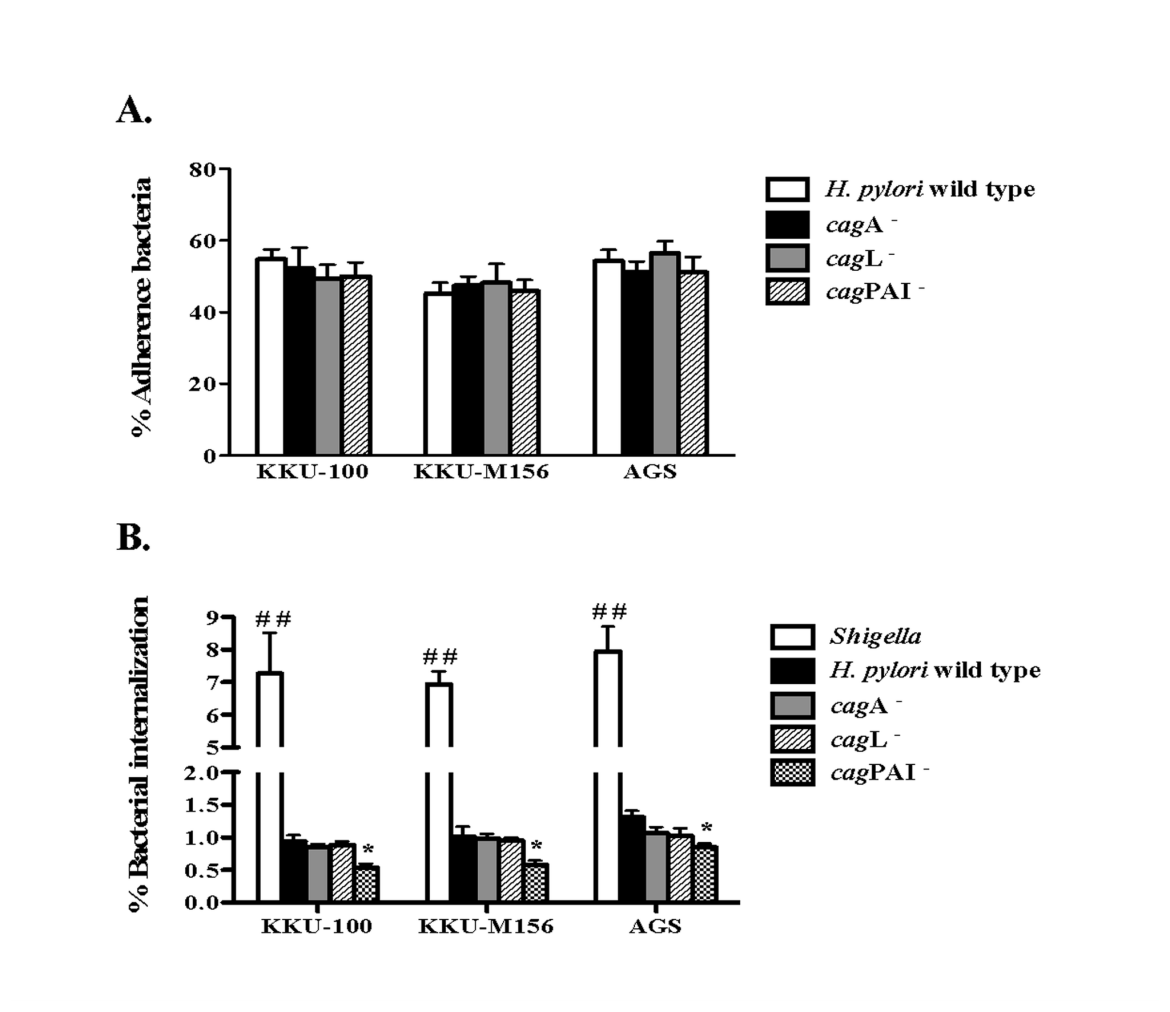
*H. pylori* adhesion and internalization. **A**. **Percentage of *H. pylori* adhesion and B. internalization in biliary (KKU-100 and KKU-M156) and gastric (AGS) cells incubated with *H. pylori* wild type, *cagA*^-^, *cagL*^-^ or *cag*PAI^-^ mutant strains for 6 h**. *H. pylori* adhesion or internalization was determined by bacterial culture and interpreted as the percentage adherence or internalization compared with the starting number of *H. pylori*. Data represent the mean ± SEM in triplicate experiments. * *p* < 0.05 confirmed a significant difference between the *H. pylori* wild type internalization and *cag*PAI^-^ internalization. ^##^ *p* < 0.01 indicated a significant difference between *Shigella* internalization and *H. pylori* wild type internalization.

Adhesion of *H. pylori* to biliary tract cells was measured after 6 h of co-culture with the CCA cell lines, KKU-100 and KKU-M156. No significant differences in adherence to these cell lines, or to AGS cells, was observed for the wild-type, cagA **^-^**, *cag*L**^-^** or *cag*PAI**^-^** strains (*p*>0.05) (Figure **1A**).

Figure **1B** shows *H. pylori* internalization in biliary tract epithelial cells at 6 h after co-culture. Approximately 1% of *H. pylori* bacteria invaded KKU-100 and KKU-M156 cell lines, just as we had observed in the AGS cell line (Figure 1B). As a positive control for these assays, we used the highly invasive bacterium, *S. flexneri*. Interestingly, only the *cag*PAI mutant strain had a decreased percentage of internalization in these three cell lines, with a significant difference compared with the wild type strain (*p*<0.05). Collectively, these findings suggest that although loss of the *cag*PAI does not have an effect on the ability of *H.  pylori* to adhere to CCA cells, it may affect the ability of *H. pylori* to invade these cells. 

**Figure 2:**
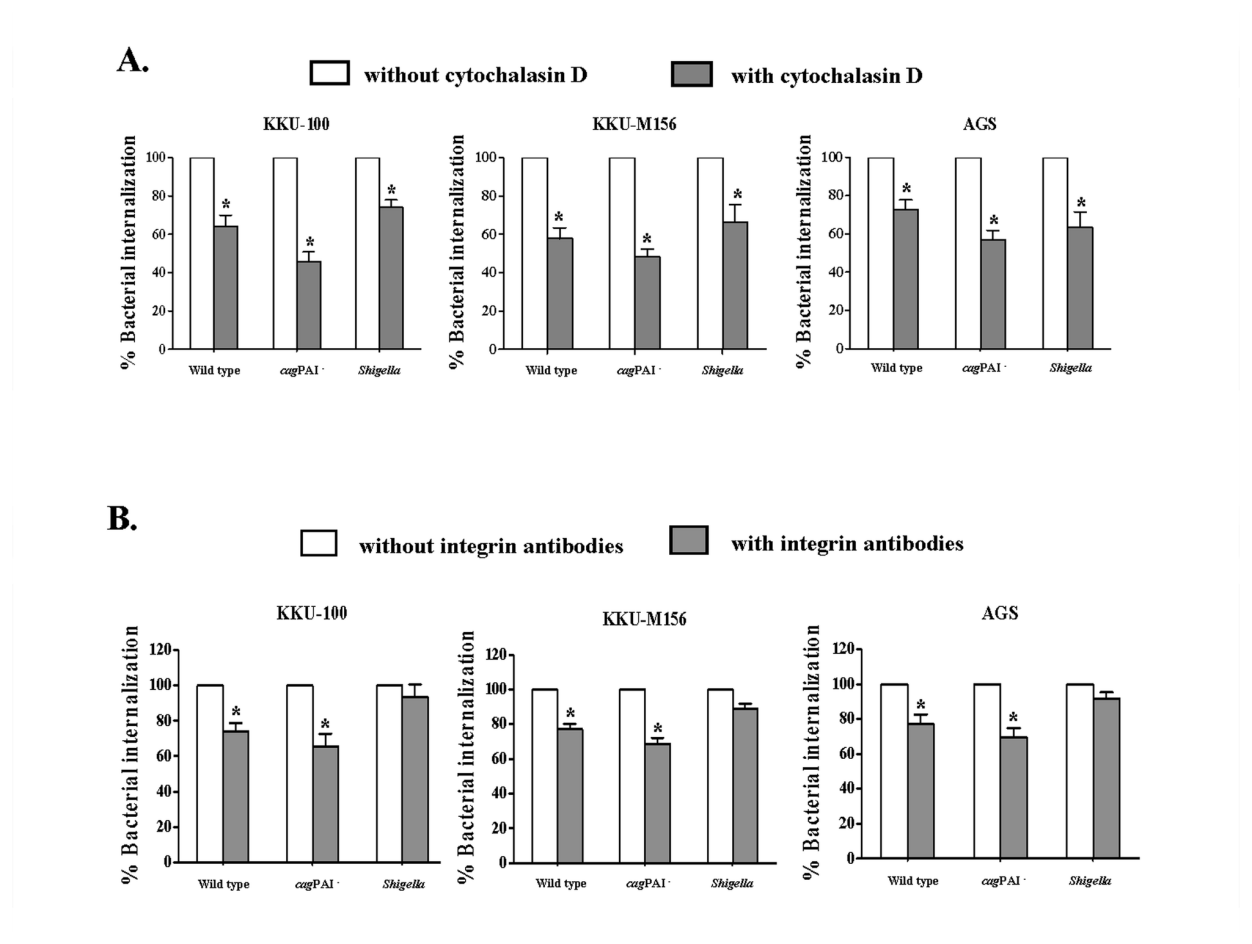
Actin polymerization and *H. pylori* internalization. **A**. **Effect of cytochalasin D (actin polymerization inhibitor) on *H. pylori* internalization in biliary (KKU-100 and KKU-M156) and gastric cells (AGS)**. After treatment with cytochalasin D, cells were incubated with *H. pylori* wild type, *cagA* or *cag*PAI mutant strains for 6 h. The *H. pylori* internalization was assessed by bacterial culture. The percentage of *H. pylori* internalization in cytochalasin D-treated cells was compared to the number of *H. pylori* internalization in untreated control cells**. B**. **Effect of α5β1 integrin antibodies on *H. pylori* internalization in biliary (KKU-100 and KKU-M156) and gastric (AGS) cells**. After pre-treatment with α5β1 integrin antibodies, cells were incubated with *H. pylori* wild type, cagA **^-^** or *cag*PAI**^-^** strains for 6 h. *H. pylori* internalization was accessed by bacterial culture. The percentage of *H. pylori* internalization in α5β1 integrin-antibody-treated cells was compared to the number of *H. pylori* internalization in untreated control cells. Data are the mean ± SEM of triplicate experiments. * *p* < 0.05 represented a significant difference compared between cytochalasin D or α5β1 integrin antibody-treated cells and untreated cells.

We next determined the role of actin polymerization in *H. pylori* internalization, using the actin polymerization inhibitor, cytochalasin D. After treatment with this inhibitor, we observed a decrease in the percentages of internalized wild-type and *cag*PAI^-^ *H. pylori*, as well as of *S. flexneri*, in all three cell lines (KKU-100, KKU-M156, AGS), compared to the percentage of intracellular bacteria contained within untreated cells (*p*<0.05) (Figure **2A**)**.**

As integrins have been implicated in the internalization of certain intracellular bacteria, such as *Yersinia* spp. [24], we investigated the role of integrin-mediated internalization of *H. pylori* in biliary tract epithelial cells. Pre-treatment of these cells with combined anti-α5 and -β1 integrin antibodies was associated with modest but significant inhibition of intracellular wild-type and *cag*PAI^-^ *H. pylori*, compared with untreated cells (*p*<0.05) (Figure **2B**). No significant effect was observed in the antibody-treated cells that had been co-cultured with *S. flexneri* (Figure **2B**). We suggest that actin polymerization and α5β1 integrins may be required for *H. pylori* internalization in biliary cells, as has been reported for hepatocytes by Ito *et al.* [25]. 

### *H. pylori* activates NF-κB and IL-8 production in biliary tract cells

**Figure 3:**
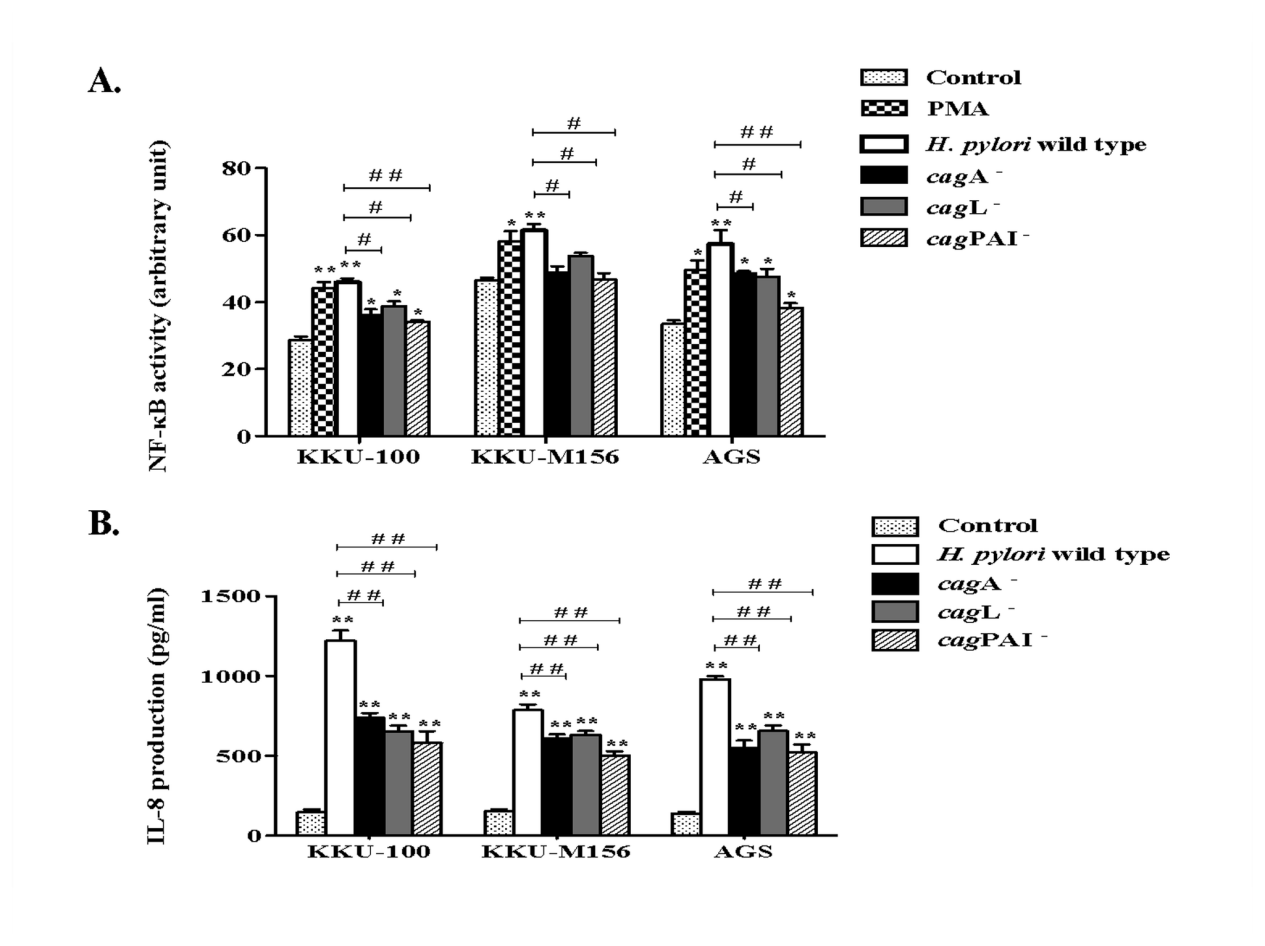
*H. pylori* activates NF-κB and IL-8 production in biliary cells. **A**. **NF-κB activation and B. IL-8 production in biliary cells (KKU-100 and KKU-M156) and gastric (AGS) cells after stimulation with *H. pylori* wild type, cagA^-^, *cag*L^-^ or *cag*PAI^-^ strains for 6 h**. NF-κB activation was measured by a Cellomics Array Scan^TM^ to measure the intensity of NF-κB translocation to the nucleus. PMA was used as a positive control. IL-8 production was determined by ELISA. The mean ± SEM of triplicate experiments are presented. * *p* < 0.05, ** *p* < 0.01 indicate a significant difference between the control cells and *H. pylori*- or PMA-treated cells. ^#^ *p* < 0.05, ^##^ *p* < 0.01 represents a significant difference between the *H. pylori* wild type-stimulated cells and cagA ^-^, *cag*L^-^ or *cag*PAI^-^-stimulated cells.

*H. pylori* strains with a functional T4SS, encoded by the *cag*PAI, are known to induce NF-κB-dependent IL-8 responses in gastric epithelial cell lines. We therefore sought to determine the ability of these bacteria to induce NF-κB activation and IL-8 production in biliary tract epithelial cells using a High Content Screening technique and ELISA, respectively. We demonstrated that wild-type *H. pylori* with a functional T4SS was able to up-regulate the levels of nuclear NF-κB translocation (Figure **3A**) and IL-8 production (Figure **3B**) in biliary tract epithelial cells. As hypothesized, similar results were observed in AGS cells (Figure **3**). Significantly higher responses were also noted in all cell types stimulated with *H. pylori* wild-type bacteria, compared with those stimulated with cagA ^-^, *cag*L^-^ or *cag*PAI^-^ strains. This is the first report of the observation that *H. pylori* requires a functional T4SS for the activation of NF-κB, leading to the production of IL-8, in biliary tract epithelial cells.

### α5β1 integrin antibodies inhibit IL-8 production in biliary cells infected with *H. pylori*

**Figure 4:**
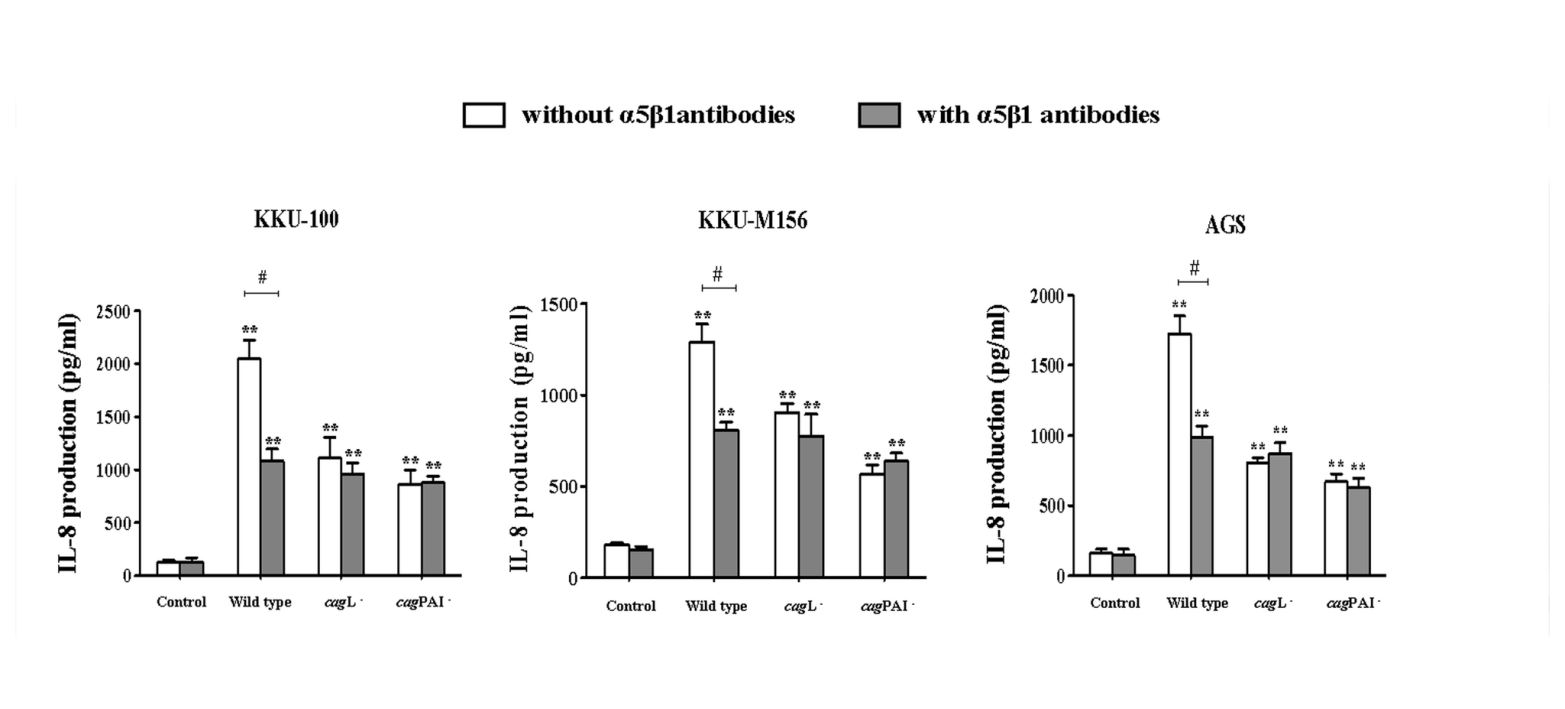
α5β1 integrins are involved in IL-8 production in biliary cells stimulated with *H. pylori*. **Combined α5β1 integrin antibodies inhibited IL-8 production in biliary cells (KKU-100 and KKU-M156) and gastric (AGS) cells with *H. pylori* wild type, *cag*L^-^ and *cag*PAI^-^ strains**. After pre-treatment with α5β1 integrin antibodies, cells were stimulated with *H. pylori* wild type, *cag*L^-^ or *cag*PAI^-^ strains for 6 h and IL-8 levels were measured by ELISA. ** *p* < 0.01 indicates a significant difference between the control cells and *H. pylori*-treated cells. ^#^ *p* < 0.05 indicates a significant difference between cells having undergone α5β1integrin antibody pre-treatment and cells that did not.

*H. pylori* CagL was reported to interact with α5β1integrins, thereby activating a downstream signaling cascade and cytokine production in host cells [15]. In order to determine whether CagL-α5β1integrin interactions are involved in *H. pylori*-mediated IL-8 responses in biliary tract epithelial cells, we pre-treated these cells with antibodies directed against α5 and β1 integrins, as previously described [23]. The levels of IL-8 production were significantly decreased in KKU-100 and KKU-M156 cells that had been pre-treated with these antibodies, when compared with untreated cells (Figure **4**). AGS cells were included as a positive control for this experiment. These results indicate that *cag*PAI-dependent *H. pylori* interactions with α5β1 integrin are involved in IL-8 production in biliary tract epithelial cells. 

### Effects of *H. pylori* on *NOD1*, *TLR2*, *TLR4* and *TLR5* gene expression in biliary cells

**Figure 5:**
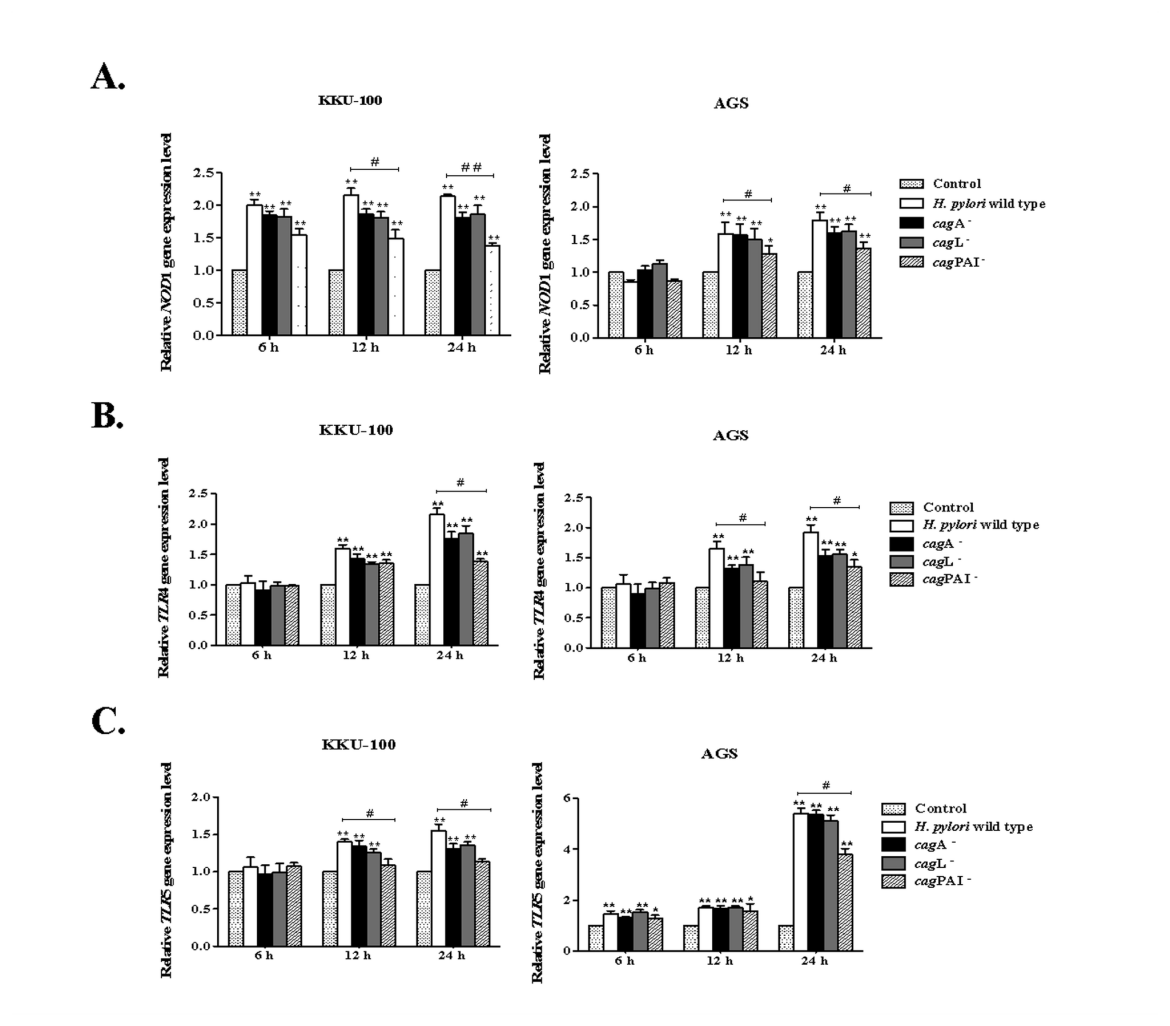
*H. pylori* up-regulates *NOD1*, *TLR4* and *TLR5* gene expression in biliary cells. **A**. ***NOD1*, B. *TLR4* and C. *TLR5* gene expression in KKU-100 and AGS cells after stimulation with *H. pylori* wild type, cagA^-^, *cag*L^-^ or *cag*PAI^-^ strains for 6, 12 and 24 h**. Gene expression was accessed by qRT-PCR. Data are presented as the mean ± SEM of triplicate experiments. * *p* < 0.05, ** *p* < 0.01 represent a significant difference between control, non-stimulated cells and *H. pylori*-stimulated cells. ^#^ *p* < 0.05, ^##^ *p* < 0.01 represent a significant difference between *H. pylori* wild type- and *cag*PAI^-^ -stimulated cells.

This study aimed to determine whether *H. pylori* could up-regulate expression of the genes encoding key bacterial-sensing PRMs in epithelial cells: *NOD1*, *TLR2*, *TLR4* and *TLR5*. For this, KKU-100 and AGS cells were treated with *H. pylori* strains (wild type cagA ^-^, *cag*L^-^ and *cag*PAI^-^) at 6, 12 and 24 h, then analyzed by quantitative RT-PCR. Although we were unable to detect *TLR2* expression in either KKU-100 or AGS cells (data not shown), gene expression levels of *NOD1* (Figure **5A**), TLR4 (Figure **5B**) and TLR5 (Figure **5C**) were significantly up-regulated in both cell types treated with *H. pylori* strains, compared with untreated cells. A comparison between the wild type and the *cag* mutant strains, especially *cag*PAI^-^ strain revealed significant decreases in the expression of these three genes in KKU-100 and AGS cells exposed to the *H. pylori cag*PAI^-^ strain. These results suggest that the presence of a *cag*PAI in *H. pylori* might be associated with up-regulated *NOD1*, *TLR4* and *TLR5* gene expression in biliary cells.

### *H. pylori* induces IL-8 production in biliary cells through NOD1 and TLRs

**Figure 6:**
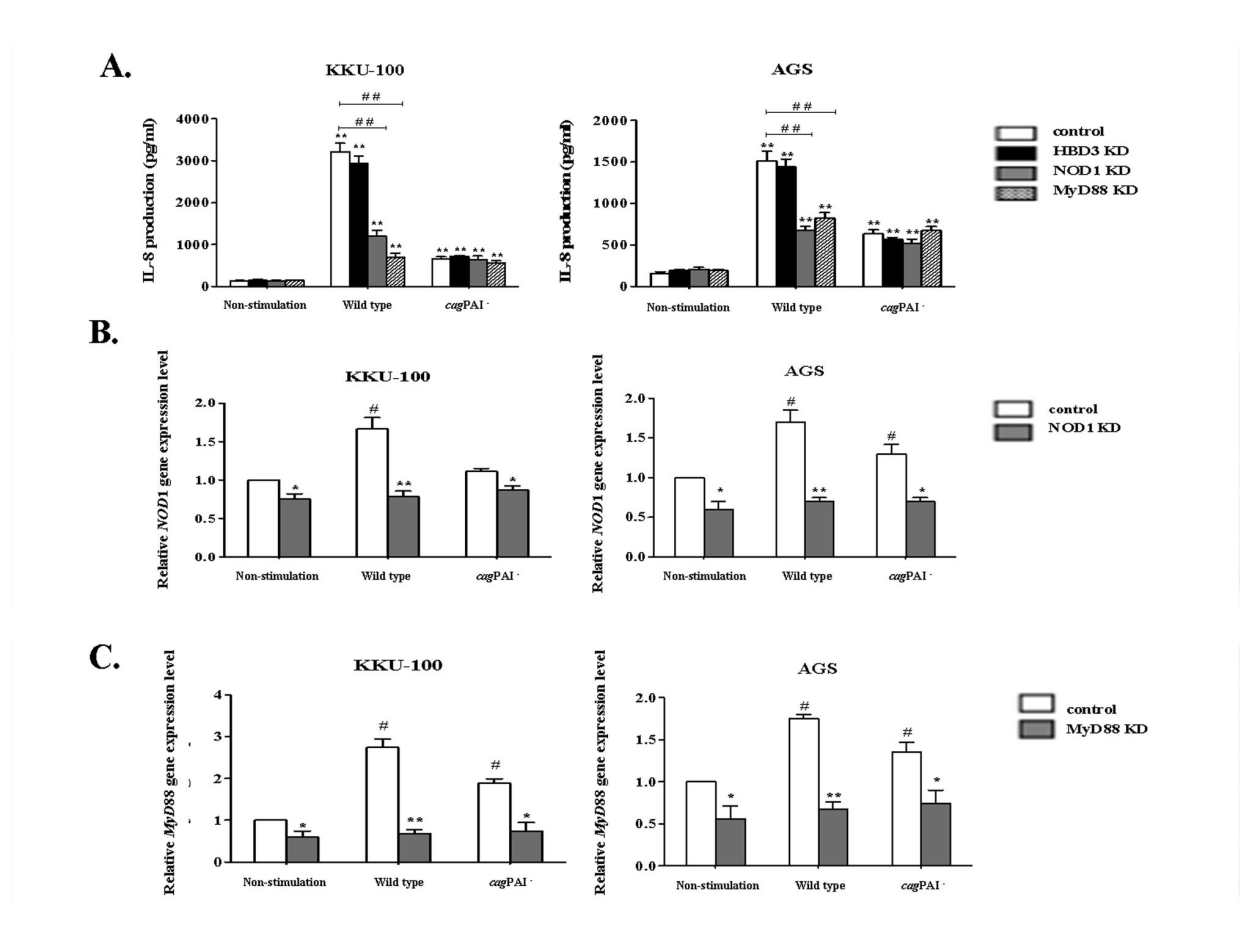
*H. pylori* induces IL-8 production in biliary cells through NOD1 and MyD88 signaling pathways. **A**. **IL-8 production, B. *NOD1* and C. *MYD*88 gene expression in KKU-100 cell line and AGS cells treated with NOD1 or MyD88 siRNA then stimulated for 24 h with *H. pylori* wild type or *cag*PAI^-^ strains**. Cells transfected with siRNA to the HBD3 gene (*DEFB10*3) were used as a negative control. Cells without bacteria are indicated as non-stimulated. IL-8 production was determined by ELISA. Data are presented as the mean ± SEM of triplicate experiments. ** *p* < 0.01 represents significant differences between non-stimulated and *H. pylori*-stimulated cells. ^##^ *p* < 0.01 represents a significant difference between the control and NOD1 or MyD88 siRNA-treated cells. Data are presented as the mean ± SEM of triplicate experiments. ^#^ *p* < 0.05 represents a significant difference between the non-stimulated and *H. pylori*-treated cells (white bar). * *p* < 0.05, ** *p* < 0.001 represents a significant difference between the control and NOD1 or MyD88 siRNA-treated cells.

*H. pylori* strains that possess a functional T4SS have been reported to induce NF-κB activation and IL-8 production in gastric epithelial cells via either of the innate immune signaling molecules, NOD1 or MyD88 [26,27]. While NOD1 is known to respond specifically to Gram-negative peptidoglycan, MyD88 is a co-adaptor molecule that is involved in the transduction of signals from key bacteria-sensing TLRs (e.g. TLR4 and TLR5). For these reasons, as well as the fact that these PRMs appear to be expressed in biliary tract epithelial cells (Figure **5**), we transfected KKU-100 biliary cells with siRNA directed to either *NOD1* or *MYD*88, then measured the IL-8 responses induced by *H. pylori* wild-type or *cag*PAI^-^ bacteria in these cells (Figure **6A**).

AGS cells were also transfected with these siRNA. (siRNA knock-down of NOD1 or *MYD*88 gene expression in these cells was confirmed by qRT-PCR) After transfection with the appropriate siRNA, the expression levels of *NOD1* and *MYD88* in *H. pylori*-stimulated cells were significantly decreased by 60% and 70%, respectively, in KKU-100 and AGS cells, compared with non-transfected control cells (Figure **6B and 6C**). IL-8 responses in the KKU-100 cells—in which either *NOD1* or *MYD*88 gene expression had been knocked-down prior to 24 h-stimulation with *H. pylori* wild-type bacteria—were decreased by 50-70% compared with the KKU-100 control cells or cells that had been transfected with an irrelevant siRNA (to the HBD3 gene, *DEFB103*) (Figure **6A**). Similar findings were observed for AGS cells.

According to previous findings [21] and the results of our own work, *H. pylori cag*PAI^-^ bacteria induced significantly reduced IL-8 responses in both KKU-100 and AGS cells. It appears, therefore, that *H. pylori* bacteria encoding a functional T4SS are able to induce IL-8 production in biliary tract epithelial cells in a NOD1- and MyD88-dependent manner. 

## Discussion

Several reports have described the association of *Helicobacter* spp. with hepatobiliary diseases, particularly *H. pylori* and hepatobiliary cancer [2,4,28]. We previously reported the prevalence of *H. pylori* in CCA patients and that this was associated with biliary inflammation and proliferation [6]. These findings suggested that *H. pylori* may be playing a causal role in the pathogenesis of hepatobiliary diseases, however, there has been limited information regarding the effect of *H. pylori* bacteria on hepatobiliary cells. While *H. pylori* adhesion and internalization in biliary tract epithelial cells has been reported [25], it was not determined whether *cag*PAI-encoded factors were required for these processes. 

In the current study, the ability of *H. pylori* to adhere to and invade biliary cells was determined. The ability of *H. pylori* to adhere and be internalized by biliary cells (KKU-100 and KKU-M156) was similar to that seen in AGS cells. We found that cagA and *cag*L mutations had no effect on *H. pylori* adhesion and internalization, compared with the wild type bacteria, whereas the *cag*PAI appears to be required for *H. pylori* entry into cells (Figure **1B**). These findings are similar to a previous report that showed that an *H. pylori* cagA mutant strain was unaffected in its ability to adhere to and invade AGS cells [29]. 

The *cag*PAI has been shown to exert multiple effects on infected cells, including cytokine production, actin polymerization, disruption of cell-to-cell junctions and altered cell proliferation [30]. It has been proposed that actin polymerization is involved in the internalization of *H. pylori* by AGS cells [31,32]. We performed studies to elucidate the role of actin polymerization and integrins in *H. pylori* internalization and responses in CCA and AGS cells and found that the *H. pylori cag*PAI mutant strain is indeed associated with decreased bacterial internalization in biliary cells (KKU-100 and KKU-M156) and AGS cells, when compared with the wild type. 

Integrins are transmembrane glycoproteins that mediate cell-cell, cell-extracellular matrix and cell-pathogen interactions [33]. Integrins are involved in the transduction of many forms of signals to cells, including proliferation, differentiation, survival, control of transcription and actin polymerization [34]. The *H. pylori* CagL protein contains RGD-motifs, shown to be important for interaction with β1 integrin [10]. To investigate the role of actin polymerization in *H. pylori* internalization, biliary cells and AGS cells were treated with cytochalasin D (an actin polymerization inhibitor) or α5β1 integrin blocking antibodies, prior to *H. pylori* stimulation. After blocking actin polymerization in biliary cells, we observed a decrease in the internalization of both wild type and *cag*PAI^-^ *H. pylori* strains. The data indicated that the effect of *cag*PAI (T4SS) might be involved in *H. pylori* internalization in both biliary and gastric cells. We speculate that actin polymerization and α5β1 integrin signaling might be involved for *H. pylori* internalization in the biliary cells. These results are similar to those of Ito and colleagues who showed that actin polymerization and the β1 integrin receptor were required for *H. pylori*-internalization in hepatocyte cells [25]. The role of the *H. pylori cag*PAI in internalization in hepatobiliary cells, however, remains unclear and requires further study. Internalization or invasion of *H. pylori* into host cells is considered a mechanism for escaping host immune responses [35]. *H. pylori* capable of invading both epithelial (AGS) and immune cells (macrophages) have been reported [32,35]. The current study suggests that the role of *cag*PAI might be involved in immune evasion by *H. pylori* in the hepatobiliary system.

*H. pylori* cagA and the T4SS, encoded by the *cag*PAI, are involved in NF-κB activation and induction of IL-8 production [9]. IL-8 is a potent chemokine that mediates the recruitment and activation of neutrophils [36], associated with severe gastritis [37]. Inactivation of the genes contained in the *cag*PAI results in decreased activation of NF-κB and MAPK signaling, which leads to a decrease in IL-8 production [38–40]. Backert and Naumann reviewed at least 12 different signaling pathways to activate NF-κB in gastric epithelial cells by T4SS-dependent and CagA-dependent or independent pathways, as well as by T4SS-independent effectors [41]. 

In our study, we show that wild type *H. pylori* significantly activates NF-κB and stimulates IL-8 production in biliary (KKU-100 and KKU-M156) and AGS cells. NF-κB activation and IL-8 production in these three cell lines were also significantly decreased when stimulated with *H. pylori* cagA, *cag*L and *cag*PAI mutant strains. These data indicate that *H. pylori* could promote inflammation through stimulation of IL-8 production in biliary cells in a cagPAI-dependent manner. The levels of NF-κB activation and IL-8 production were, however, similar in cells stimulated with either cagA or *cag*PAI mutant strains. Our results are in contrast to those of previous studies showing that IL-8 production in gastric epithelial cell lines was dependent on the presence of a cagPAI, but not CagA [42–44]. One explanation may be that the cagA mutant strain in the present study carries a secondary mutation in another *cag*PAI gene essential to T4SS functionality. 

Another important observation from our study was the residual levels of IL-8 production in biliary cells stimulated with *H. pylori* cag mutant strains (Figure 3B), suggesting a potential role for T4SS-independent mechanisms. One such mechanism may involve the *H. pylori* outer membrane protein, OipA, which was reported to be involved in the activation of the signal inducer and activator of transcription 1 (STAT1) cascade [45]. Further studies are required to identify the contribution of this pathway on IL-8 production in biliary cells stimulated with *H. pylori* bacteria.

Shaffer *et al.* showed that IL-8 production was significantly decreased in AGS cells infected with *H. pylori cag*L mutant bacteria compared to a *H. pylori* wild type strain, but they did not address CagL-integrin interactions [46]. The involvement of α5β1 integrin and IL-8 production in AGS cells infected with *H. pylori* was also reported [23]. In addition, it was recently reported that the T4SS machinery can induce IL-8 production via CagL-α5β1 integrin interactions and subsequent activation of MAPKs and NF-κB [15]. To investigate the role of CagL and integrin in stimulating IL-8 production in biliary cells, KKU-100, KKU-M156 and AGS cells were treated with α5β1 integrin antibodies before *H. pylori* stimulation. After stimulation with *H. pylori*, a decrease in IL-8 production was found in biliary (KKU-100 and KKU-M156) and gastric (AGS) cells treated with α5β1 integrin antibodies compared with untreated cells. These results suggest that CagL and integrin might be involved in IL-8 production in biliary cells, as previously reported in AGS cells [15,46].

*H. pylori* exploits integrin for its pathogenesis [10]. It has also been reported that *H. pylori* induces α5 and β1 integrin expression in the AGS cell line and that Ras, AP-1 and NF-κB were found to be involved in the expression of α5 and β1 integrins [47]. In the current study, the expression of α5 and β1 integrin in biliary cells was also investigated and it was found that cagA, *cag*L and *cag*PAI were all required for α5β1 integrin expression in biliary cells (data not shown). These results are similar to a previous report by Zhang and colleagues who used proteomic analysis to demonstrate that *H. pylori* induced up-regulated β1 integrin expression in human hepatic cells (HepG2) [48]. Another report suggested that the excessive expression of integrins may be involved in tumor progression, including cell invasion, metastasis, angiogenesis, cell transformation and cell proliferation [33]. We further hypothesize that the *cag*PAI of *H. pylori* accelerates CCA progression by signaling via integrins. 

The cytosolic innate immune protein, NOD1, plays a role in host defense against microbial infection [49]. TriDAP, a component of microbial peptidoglycan, is recognized by NOD1, promoting inflammatory cytokine responses [26]. Recently, a previous report in gastric epithelial cells showed that *NOD1* gene expression was up-regulated in response to exposure with *H. pylori* and that these responses occurred in a *cag*PAI-dependent manner [21]. Consistent with that observation, we found that *H. pylori* could up-regulate *NOD1* gene expression in a *cag*PAI-dependent manner in both biliary and gastric cell lines. 

In addition to NOD1, other innate immune molecules of the TLR family have been shown to be involved in pro-inflammatory cytokine responses to microbial infection [27]. TLR2, 4 and 5 have all been reported to be involved in the recognition of *H. pylori* [50–52]; nonetheless, these findings remain controversial. In the current study, we were unable to detect *TLR2* expression by real-time PCR in biliary and gastric cell lines, thus further analysis of *TLR2* was not performed. An increased expression level of *TLR4* and *TLR5* genes were, however, detected in biliary and gastric cells after stimulation with *H. pylori*, which was dependent on the presence of a *cag*PAI. These results are consistent with a previous study that showed that the lipopolysaccharide of *H. pylori cag*PAI^+^ strains induced *TLR4* expression in guinea pig gastric pit cells [50]. These findings, though, contrast with those from another study that showed that *TLR4* gene expression increased following *H. pylori* infection, in a *cag*PAI-independent manner [52]. This difference might be the result of different strains of *H. pylori* or the multiplicity of infection (MOI) used in each study. Thus, the data suggest that the *cag*PAI-encoded T4SS of *H. pylori* may be involved in initiating inflammatory responses in biliary cells via up-regulation of *NOD1*, *TLR4* and *TLR5* gene transcription. Further studies are required to confirm these findings. 

In order to investigate the roles of NOD1 and TLRs on IL-8 production in biliary cells stimulated with *H. pylori*, we pre-treated cells with siRNA to the respective genes. We found reduced levels of IL-8 production in *H. pylori*-stimulated biliary and gastric cells that had been pre-treated with either *NOD1* or *MyD*88 siRNA, when compared with cells treated with an irrelevant siRNA. Similarly, IL-8 production was markedly impaired in AGS NOD1 knock-down cells stimulated with *H. pylori*, indicating that NOD1 signaling was involved in pro-inflammatory responses in *H. pylori*-stimulated cells [21]. These data are consistent with those of previous studies [14,21], as well as those of Gorrell et al. [15] who despite finding no role for NOD1 in CagL-dependent IL-8 responses, found that NOD1 contributes to T4SS-dependent IL-8 responses induced by *H. pylori* bacteria. Interestingly, in the present work, we also observed a significant effect of *MyD*88 gene knockdown on IL-8 responses in *H. pylori*-stimulated AGS cells. Indeed, a previous study reported that *MyD*88 but not *NOD1* siRNA-treated AGS cells produced significantly lower IL-8 responses to *cag*PAI-positive *H. pylori* bacteria [11]. Nevertheless, no data were presented in that study to confirm the efficacy of the NOD1 knockdown and thus the possibility of NOD1 involvement in IL-8 production could not be excluded. Further investigations are thus warranted to determine the relative contributions of MyD88 and NOD1 in *H. pylori* T4SS-dependent IL-8 responses in AGS cells. In biliary cells, we propose that both NOD1 and MyD88 signaling pathways may be required for *H. pylori* T4SS-dependent inflammation.. 

In conclusion, the present study suggests that the *cag*PAI encodes factors that may be associated with *H. pylori* internalization in biliary cells. Additionally, α5β1 integrin appeared to be involved with *H. pylori* internalization and its ability to induce IL-8 production in these cells via NOD1 and MyD88-dependent signaling pathways. Thus, the current study provides evidence for a role of *H. pylori* in the activation of pro-inflammatory signaling pathways in hepatobiliary cells, similar to that already reported in gastric epithelial cells. Further studies using animal models should be conducted to clarify the exact role of *H. pylori* in pathogenesis associated with the hepatobiliary system. 
